# Employing QbD strategies to assess the impact of cell viability and density on the primary recovery of monoclonal antibodies

**DOI:** 10.1002/elsc.202200056

**Published:** 2022-12-23

**Authors:** Ole Jacob Wohlenberg, Carlotta Kortmann, Katharina V. Meyer, Thomas Scheper, Dörte Solle

**Affiliations:** ^1^ Leibniz Universität Hannover Institut für Technische Chemie Hannover Germany

**Keywords:** CHO, DoE, mAb, PAT, QbD

## Abstract

Quality by Design (QbD) is one of the most important tools for the implementation of Process Analytical Technology (PAT) in biopharmaceutical production. For optimal characterization of a monoclonal antibody (mAb) upstream process a stepwise approach was implemented. The upstream was divided into three process stages, namely inoculum expansion, production, and primary recovery, which were investigated individually. This approach enables analysis of process parameters and associated intermediate quality attributes as well as systematic knowledge transfer to subsequent process steps. Following previous research, this study focuses on the primary recovery of the mAb and thereby marks the final step toward a holistic characterization of the upstream process. Based on gained knowledge during the production process evaluation, the cell viability and density were determined as critical parameters for the primary recovery. Directed cell viability adjustment was achieved using cytotoxic camptothecin in a novel protocol. Additionally, the cell separation method was added to the Design of Experiments (DoE) as a qualitative factor and varied between filtration and centrifugation. To assess the quality attributes after cell separation, the bioactivity of the mAb was analyzed using a cell‐based assay and the purity of the supernatant was evaluated by measurement of process related impurities (host cell protein proportion, residual DNA). Multivariate data analysis of the compiled data confirmed the hypothesis that the upstream process has no significant influence on the bioactivity of the mAb. Therefore, process control must be tuned towards high mAb titers and purity after the primary recovery, enabling optimal downstream processing of the product. To minimize amounts of host cell proteins and residual DNA the cell viability should be maintained above 85% and the cell density should be controlled around 15 × 10^6^ cells/ml during the cell removal. Thereby, this study shows the importance of QbD for the characterization of the primary recovery of mAbs and highlights the useful implementation of the stepwise approach over subsequent process stages.

**Abbreviations**
CHO: Chinese Hamster Ovary
CQA: Critical Quality Attribute
CPP: Critical Process Parameter
CPT: Camptothecin
DoE: Design of Experiments
HCP: Host Cell Protein
L929 cell: Adherent mouse fibroblast L929 cell
mAb: monoclonal Antibody
MLR: Multiple Linear Regression
MVDA: Multivariate Data Analysis
PAT: Process Analytical Technology
PM: Production Medium
QbD: Quality by Design
SARS‐CoV‐2: Severe Acute Respiratory Syndrome Corona Virus 2
TCD: Total Cell Density
TNF‐alpha: Tumor Necrosis Factor alpha

## 1 INTRODUCTION

The monoclonal antibody market is growing rapidly, with expected total market revenues reaching a valuation of 300 billion US$ by 2025 [1, 2]. Due to major advancements in the biopharmaceutical industry, the number of medical applications is quickly increasing, including treatments for cancer as well as novel diseases such as the Severe Acute Respiratory Syndrome CoronaVirus‐2 (SARS‐CoV‐2) [3, 4, 5, 6]. The traditional approach for product and process development consists of a rigid manufacturing process with predefined set points and batch‐to‐batch quality controls, resulting in a lack of methodical connection between the process, product, and application. In order to enable fast approval and release to market of novel therapeutic antibodies fulfilling high‐quality standards the development and production process has to be performed in a structured and controlled environment [7].

Following this agenda, the FDA introduced a guideline to biopharmaceutical development and manufacturing with the *Current Good Manufacturing Practice for the 21st century* initiative in 2004. This protocol includes the framework for Process Analytical Technology (PAT) and guidelines from the International Conference of Harmonisation, which introduced the concept of Quality by Design (QbD) as a risk‐based approach to the development of new therapeutics [8, 9, 10, 11, 12]. This approach defined the main objective of research as a way to construct and methodically build quality into the process and product during the development phase instead of testing it into the product during production. Thereby biopharmaceutical manufacturers are able to tune their processes toward product quality in a compliant environment, resulting in improved flexibility, cost reduction, and faster adjustments in production as well as the development [13, 14].

Quality by Design is structured as a multi‐step process, starting with the identification of process parameters and a risk assessment, followed by a Design of Experiments (DoE) approach to examine critical process parameters (CPPs) regarding critical quality attributes (CQAs) [15, 16]. CPPs are evaluated process inputs like the initial viable cell density or the culture pH, while CQAs include process and product outputs like the growth rate of the cells or the bioactivity of the produced antibody. The systematic set up of the DoE enables the investigation of factor effects and interactions, which can be statistically solidified using multivariate data analysis (MVDA). Based on the data and the mathematical model a designated design space can be calculated, representing a multi‐dimensional region of process parameters in which the process can be conducted within predefined quality attributes [9, 17]. Thereby, working within the set design space is not considered to be a change or risk to the process or product quality.

In order to assess the complete upstream process of a mAb production using Chinese Hamster Ovary (CHO) cells the process was split up into steps, which were investigated individually. Thereby, this work was based on the previous findings regarding the inoculum expansion and production process and marks the final step of the complete case study process characterization [18, 19]. Even though the presented QbD strategies are widely used in the biopharmaceutical industry, the split‐up approach to process characterization was rarely implemented [20, 21]. This approach enhances the analysis by connecting intermediate quality attributes to following process steps, allowing for a holistic assessment of the process risks and robustness.

By that, gained knowledge about factor effects on intermediate CQAs can be used to define the setup of the following experiments. Earlier studies showed no significant impact of the production process on the investigated product quality elucidating the mAb quantity and purity as the main quality criteria for the production step [19]. Furthermore, they showed a strong influence of the culture pH, pO_2_, and the initial viable cell density on the viability in correlation with the proportion of produced mAb to process‐related impurities [19]. Therefore, this work will focus on viability as an input factor for the first step of cell removal. The directed adjustment of the cell viability for analysis in a DoE approach was established by the use of camptothecin (CPT), which has a planar pentacyclic ring structure and acts as a topoisomerase inhibitor, resulting in a cytotoxic effect on the CHO cells [22]. This enables the viability adjustment for the first time without altering the culture duration or variation of other process parameters. Critical quality attributes like the bioactivity of the produced antibody as well as the amount of residual DNA and host cell proteins (HCPs) in the supernatant were analyzed to establish a robust design space for the last step of the upstream process.

## 2 MATERIAL AND METHODS

Presented QbD strategies will be implemented in the primary recovery of an IgG1 monoclonal antibody (mAb) production process using a DG44 CHO cell line (Sartorius Stedim Cellca GmbH, Germany).

### 2.1 Cell line and material

The first steps of the process, namely the vial thaw and inoculum expansion were performed as described by Boehl et al. [18]. The production process step was performed in the modular Ambr®250 system (Sartorius, Germany), using proprietary and chemically defined production medium (PM) and two additional feed media (feed medium A; feed medium B) for macro nutrients (e.g., glucose) and micro nutrients (e.g., amino acids) respectively (Sartorius Stedim Cellca GmbH, Germany) [23]. One Ambr®250 vessel was used to cultivate the cells for the DoE, while the second vessel was used as an internal reference standard.

The cultivation was conducted over 9 days with daily feeds (1% feed medium A, 0.1% feed medium B) from day 3 and additional glucose feeds (400 g/L stock solution) to a culture glucose concentration of 5 g/L from day 5. At peak cell density from day 7 to 9 the cells were cultivated in 250 ml shake flasks (Corning, USA) in a Heracell 240 CO_2_ incubator at 36.8°C and 7.5% CO_2_ (Thermo Scientific, USA) using a MaxQ CO_2_ Plus shaker platform (Thermo Scientific, USA).

**PRACTICAL APPLICATION**

Consistent adjustment of critical process parameters is the key for meaningful DoE analysis. Therefore, cytotoxic camptothecin was used in a novel approach to adjust the cell viability at peak cell densities. Thereby, the viability can be used as an input factor for the process characterization, enabling further analysis of the effects and interactions of cell viabilities without elongation of the process duration or variation of other process parameters. This highlights the importance of novel strategies to implement QbD principles on various process steps.

### 2.2 Directed cell viability adjustment

The viability adjustment was conducted in shake flasks to treat cells from a single process. Thereby, improving the comparability of the DoE runs, compared to using the two available Ambr®250 vessels for multiple cultivations. After transfer to the shake flasks, camptothecin was added to concentrations of 10 and 30 μM to adjust the targeted cell viabilities of ∼60% and ∼80%, respectively. The used concentrations were evaluated by a standard series of camptothecin at peak viable cell density (20 × 10^6^ cells/ml). Viabilities for the multivariate data analysis were calculated by the combination of the relative decline in cell density to the reference cultivation and the measured viability for each cultivation run.

### 2.3 Analytics

During the production process 1 ml samples were taken daily. Viable cell densities and viabilities were measured using a Cedex HiRes (Roche Innovatis, Switzerland). The pH (7.2) and pO_2_ (60%) were measured and controlled by the Ambr®250 system. Offline pH measurements for offset calibration (for ΔpH > 0.05) were performed using a FiveEasy Plus pH meter FP20‐Micro (Mettler Toledo, USA) every 2 days.

Substrates (glucose, lactate, glutamine, glutamate), the produced mAb and total protein concentrations were analyzed during the production process and after the cell separation using the Cedex Bio (Roche, Switzerland). The DNA concentration in the supernatant was analyzed using the Nanodrop 2000 (Thermo Scientific, USA). Antibody bioactivity was determined in triplicates by an adherent mouse fibroblast (L929) cell (CLS Cell Lines Service, Germany; catalog number 400260) based assay using the tumor necrosis factor alpha (TNF‐α) under the presence of actinomycin D. L929 cell viability was analyzed using the cell titer‐blue assay (Promega, USA) after 24 h of treatment with the antigen and antibody. The produced antibody was diluted and used in low and high concentrations of 8 and 80 ng/ml, respectively. The antigen TNF‐ α was used in a fixed concentration, determined to result in around 20% L929 cell viability without addition of functional antibody.

### 2.4 Cell separation

The cell separation at the end of the process was performed by centrifugation and filtration of 6 ml cell culture broth for each experimental run. Centrifugation was performed for 5 min at 300 × *g* using a Centrifuge 5702 (Eppendorf, Germany), while filtration was performed using Sartoclear Dynamics Lab P15 syringes with 0.2 μm filters (Sartorius, Germany).

### 2.5 Design of experiments (DoE)

The design and analysis was performed using the DoE software MODDE 12 (Umetrics, Sartorius Stedim Data Analytics, Sweden). Three critical parameters determined during risk assessment were used as factors (F_1_ = viability, F_2_ = cell density, F_3_ = separation method) for full factorial designs with three center point runs. With the viability varied on three levels and the third factor as a qualitative factor, the design resulted in two full factorial approaches with two factors (F_1_ and F_2_) for each separation method with a total of 18 experiments. Hereinafter, the factor settings are described as 0 for center point level and –1/1 for the low and high levels of the full factorial squares, respectively. The qualitative factors for the separation method are abbreviated as C for centrifugation and F for filtration.

The cell viability was varied equally between 60% and 99 % by addition of camptothecin. For the cell density, the cell broth was diluted with fresh PM before cell separation. Total cell densities were varied equally between 10 and 20 × 10^6^ cells/ml. An overview of the experimental setup and the explained numerical coding of the parameter levels are depicted in the supplements. Three responses were analyzed after the cell separation: mAb proportion to impurities, residual DNA in the supernatant and product bioactivity.

### 2.6 Multivariate data analysis (MVDA)

The mathematical models were fitted using multiple linear regression (MLR) with squares and interactions in MODDE 12 (Umetrics, Sartorius Stedim Data Analytics, Sweden) as described by Boehl et al. [18]. Model statistics, namely the R‐squared, adjusted R‐squared, Q‐squared, model validity, and reproducibility were calculated to assess the conducted model. Factors with a coefficient of zero in its confidential interval are regarded to have no significant influence on the response and were therefore removed from the model. The design space was calculated using Monte Carlo simulations with parameter limits summarized in the supplements.

## 3 RESULTS AND DISCUSSION

Key objective of this study was the investigation of parameter effects on the robustness und quality of the primary recovery for the mAb production process. Several process parameters were evaluated in regard to their importance for the process and theoretical risks for further downstream processing. Identified critical process parameters were evaluated based on a DoE approach with a focus on mAb quality and process related impurities after the cell separation. Thereby, a quality focused process evaluation was enabled, resulting in defined knowledge about the different parameter effects and interactions. This can be used to change and adjust the following downstream process for optimal product recovery.

### 3.1 Risk assessment

The quality of the production process step was earlier evaluated by analysis of multiple intermediate CQAs [19]. During the production step, product of consistent quality was produced within the investigated knowledge space [19]. Thus, the product quantity and purity were established as the most important quality criteria for the subsequent downstream process. Cell viabilities, growth rates and integral viable cell concentrations were monitored as responses for cell growth and maintenance. These attributes provide critical information about a possible delay in the culture duration or low cell maintenance. Improvements in cell densities on the other hand can lead to reduced process time and production costs as well as higher mAb titers.

In order to further investigate the critical role of these intermediate attributes, the viability and the cell density were determined as critical process parameters for the following process step, namely the cell separation. In earlier studies, low viability representing suboptimal cellular condition could be correlated to higher amounts process related impurities, which have to be removed at high cost during further downstream process steps [19]. Higher cell densities were shown to impact the effectiveness and efficiency of the cell removal [24]. However, the peak cell density during the production process was determined to have an overall higher impact on the antibody titer than productivity enhancing conditions [19]. Furthermore, high values for these parameters represent the previously established quality of the production process [18, 19].

Thereby, the viability and cell density can be used as linking parameters between mAb production and downstream processing. With the aim of a broad evaluation of the primary recovery, the separation method was also determined as a critical process parameter. The interaction between the separation method and other parameters could be especially interesting to dynamically adjust the cell removal for given process parameters. Filtration and centrifugation were compared as general standards in the biopharmaceutical industry. Internal parameters within the separation methods, such as time and speed of the centrifugation or filter size for the filtration were not investigated further, since these parameters are well optimized for the used separation protocols.

### 3.2 DoE structure and implementation

**FIGURE 1:**
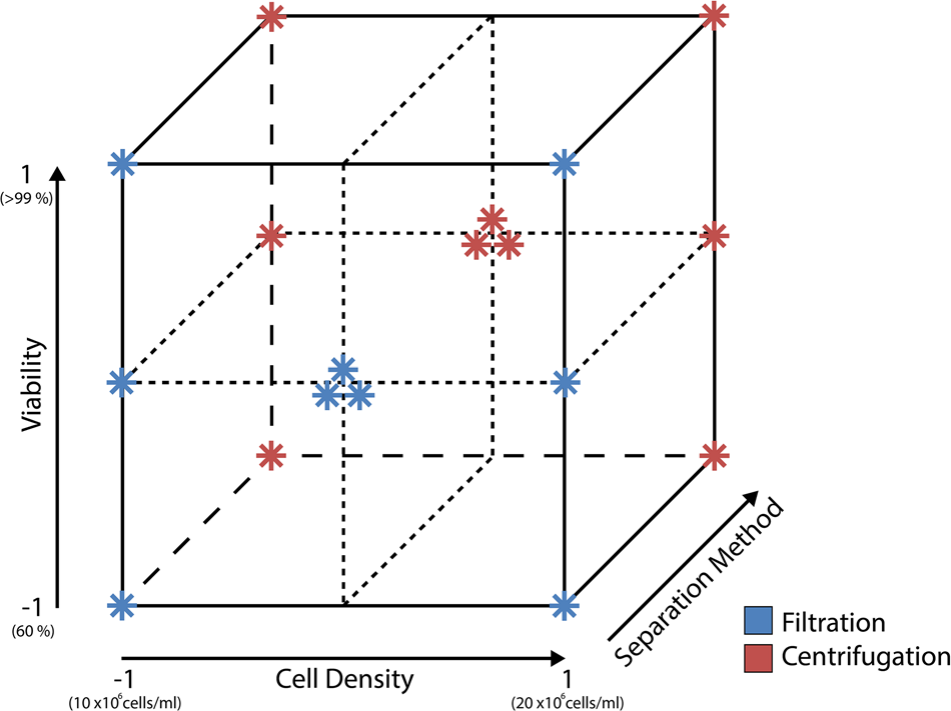
Schematic representation of the conducted design of experiments for the viability, cell density and separation method. The color of the stars represents the qualitative options for the separation method, centrifugation (red) and filtration (blue).

In order to investigate the effects and interactions of the determined critical process parameters, based on the ICH Guidelines, a DoE was set up. The factors F_1_ (viability) and F_2_ (total cell density) were combined in a full factorial design, each varied on a three‐level scale (–1, 0, 1). Since the factor F_3_ (separation method) is a qualitative factor with two options (centrifugation or filtration) there is no center level definable. Therefore, a three‐dimensional subspace was created in which the factors pattern a regular two‐level factorial design. Replicated center points were located at the centers of the front and back surfaces of the cube. By that, the quantitative factors are varied in three levels, which is desirable for the following data analysis. To further increase the available data, additional 0 level experiments were added for the viability. These were added to increase the overall data sets and comparability for the viability factor, since the method of varying CHO cell viabilities using camptothecin was newly established for this process. The resulting design added up to 18 experiments and is depicted in Figure 1.

For further in‐depth investigation of the varied process parameter effects and interactions various critical responses for the process step were determined. In order to evaluate the purity of the supernatant the residual DNA content as well as the proportion of produced antibody to process and product related impurities, like host cell proteins were analyzed. These attributes can have a severe effect on the time, yield and expenses of further downstream processing. Hence, limiting the amount of said impurities is one of the main quality aims for the upstream process and especially the primary recovery. Additionally, the bioactivity of the mAb was analyzed as its main CQA, which represents the maintenance of the mAb activity during the cell separation.

### 3.3 Directed viability adjustment

**FIGURE 2:**
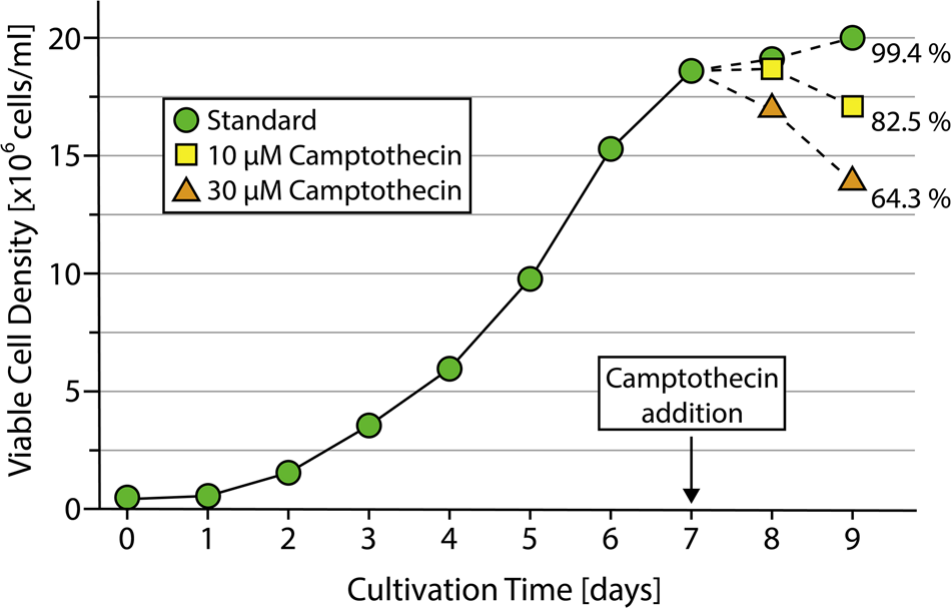
Course of cultivation with camptothecin addition on day 7. The percentage values represent the relative viability of the respective cultivation conditions.

The cell viability at the end of the culture duration was defined as one of the critical process parameters. For reliable and reproducible DoE analysis the parameters must be varied consistently on the described levels. So far, directed adjustment of cell viabilities without elongation of the culture duration was a great challenge. Longer cultivation times lead to large variances in the cell density and product titer, therefore distorting the comparability of the experimental set up. That is why camptothecin (CPT) was used in different concentrations to adjust the cell viability at the end of the exponential growth phase. The cytotoxic effect of CPT on the mAb producing CHO cells was analyzed for various concentrations, with 10 and 30 μM being used to lower the cell viability to ∼80% and ∼60% respectively over 48 h. The viable cell densities over the cultivation with addition of CPT at the beginning of the stationary phase are depicted in Figure 2.

While the cell density in the standard cultivation approached the stationary phase with around 20 × 10^6^ cells/ml, the cultures treated with CPT showed a decline in viable cell densities. This resulted in relative end viabilities of 82.5% and 64.3 % for 10 and 30 μM CPT treatments, respectively. This confirmed the effectiveness of the newly established protocol and enabled further investigation of the viability as a factor within the DoE approach.

### 3.4 Bioactivity assay

**FIGURE 3:**
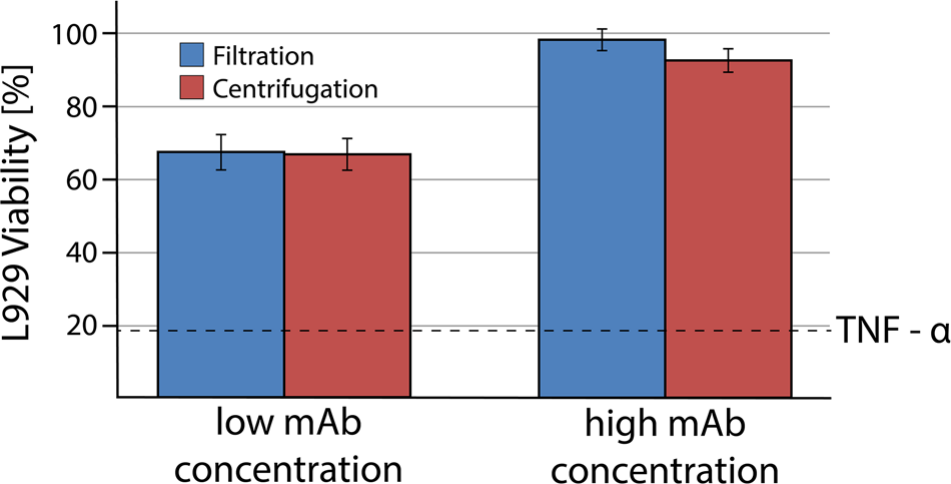
Mean results of bioactivity assay for all nine experimental runs of the filtration and centrifugation level with the dashed TNF‐α line representing cell viabilities without addition of monoclonal antibody.

Product bioactivity is one of the most important critical quality attributes for the upstream process of monoclonal antibody production. All samples generated during the experimental phase of the DoE were investigated using a cell‐based bioactivity assay, which is based on the mAb property to bind and inactivate the cytotoxic antigen TNF‐α. Thereby, the viability of the used L929 assay cells represents the bioactivity of the mAb sample. High bioactivity indicates a well working and posttranslational functionalizing antibody production. Assay results are depicted in Figure 3, with the dashed TNF‐α line representing cell viabilities without the addition of monoclonal antibody.

The shown results represent the mean bioactivities of all experimental runs (see above in Figure 1) for the filtration (blue) and the centrifugation (red) for the given mAb concentrations. Deviation between the investigated runs of each separation method as well as the overall difference between filtration and centrifugation was insignificant. The standard deviation of the center point runs was in the same range as the overall deviation for all experiments, resulting in insufficient modeling for the bioactivity as a DoE response, which confirms the consistent bioactivity over the various experimental runs. That is why the bioactivity was excluded from further multivariate data analysis. These results elucidate the hypothesis that within the investigated knowledge space the upstream process has no significant effect on the antibody bioactivity for the described process. Therefore, a high mAb titer and low amounts of process‐related impurities can be defined as the main goal for the upstream process, which were further investigated using statistical modeling.

### 3.5 Multivariate data analysis

**FIGURE 4:**
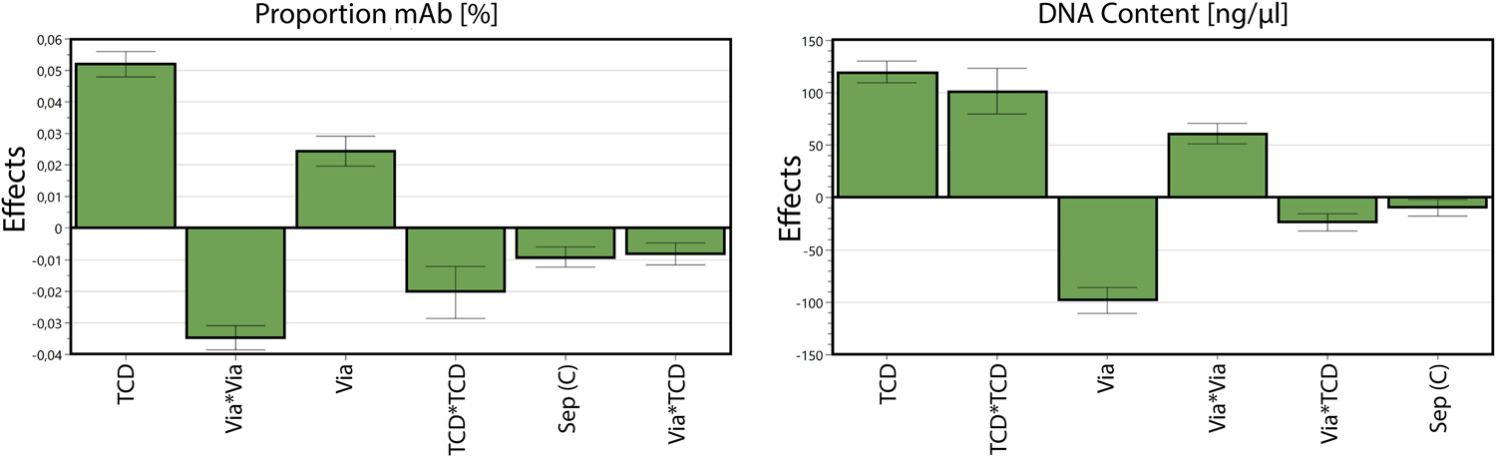
Factor effects (TCD = total cell density; Via = cell viability; Sep = separation method) for the studied responses. Factor squares and interactions are combined with a star. Only significant factors and interactions are considered and displayed in descending order for each response.

The experimental data was evaluated by calculation of a statistical model using multiple linear regression (MLR), leading to specific conclusions for factor effects and interactions for the studied parameters. Figure 4 presents the main effects plot for the mAb proportion and the residual DNA content.

The key factors total cell density and viability showed a high impact on the selected responses, both as linear effects (TCD; Via) and non‐linear effects (TCD*TCD; Via*Via). Higher cell densities during the cell separation increase the proportion of antibody as well as the residual DNA content in the supernatant. Its strong quadratic effect shows a significant non‐linearity in the correlation. The viability shows effects with positive coefficients on the proportional mAb content and effects with negative coefficients on the residual DNA. For both responses the quadratic effect highlights the non‐linear effect of the viability. In contrast, the separation methods showed only marginal to non‐significant effects and where therefore mainly excluded from the mathematical model. That means the different separation methods did not result in considerable changes in the investigated quality attributes and can be evaluated as equally effective for the first step of the primary recovery.

Accuracy of the regression models was verified by analyzing the corresponding model statistics as described by Wohlenberg et al. Thereby, the R‐squared (R2) term is the fraction of the variation of the response explained by the model, while the adjusted R‐squared (R2 adj) term is adjusted for the degrees of freedom of the analyzed model. Values over 0.5 for these terms ensure high model significance. Model accuracy of future predictions is statistically estimated by the Q‐squared (Q2) term. Values for Q2 should exceed 0.1 for significant models and 0.5 for good models. The model validity checks for diverse model problems. A value less than 0.25 predicts statistically significant model problems, such as the presence of outliers, transformation problems in the calculation, or incorrect model terms. The reproducibility compares the variation of the center point replicates to the overall variability, with a value over 0.5 insuring high model reproducibility. The calculated model statistics for the proportional mAb as well as the DNA content are summarized in Table 1.

**TABLE 1:** Summarized model statistics for the studied responses. R^2^ representing the model significance, Q^2^ representing the predictive power of the model, model validity representing possible model problems and the reproducibility representing the center point variation compared to the overall variability

	R^2^	R^2^ adj.	Q^2^	Model validity	Reproducibility
mAb proportion	0.997	0.996	0.990	0.708	0.997
DNA content	0.997	0.995	0.991	0.813	0.995

The calculated model statistics showed exceptional significance and prediction accuracy for the investigated responses with values mainly over 0.99. Model validity showed the lowest values with 0.708 and 0.813 for the mAb proportion and DNA content, respectively, which are still sufficient to rule out potential model problems. The models were also characterized as well reproducible with values close to 1, which can be explained by the small variance in the centre point runs for each cell separation method.

**FIGURE 5:**
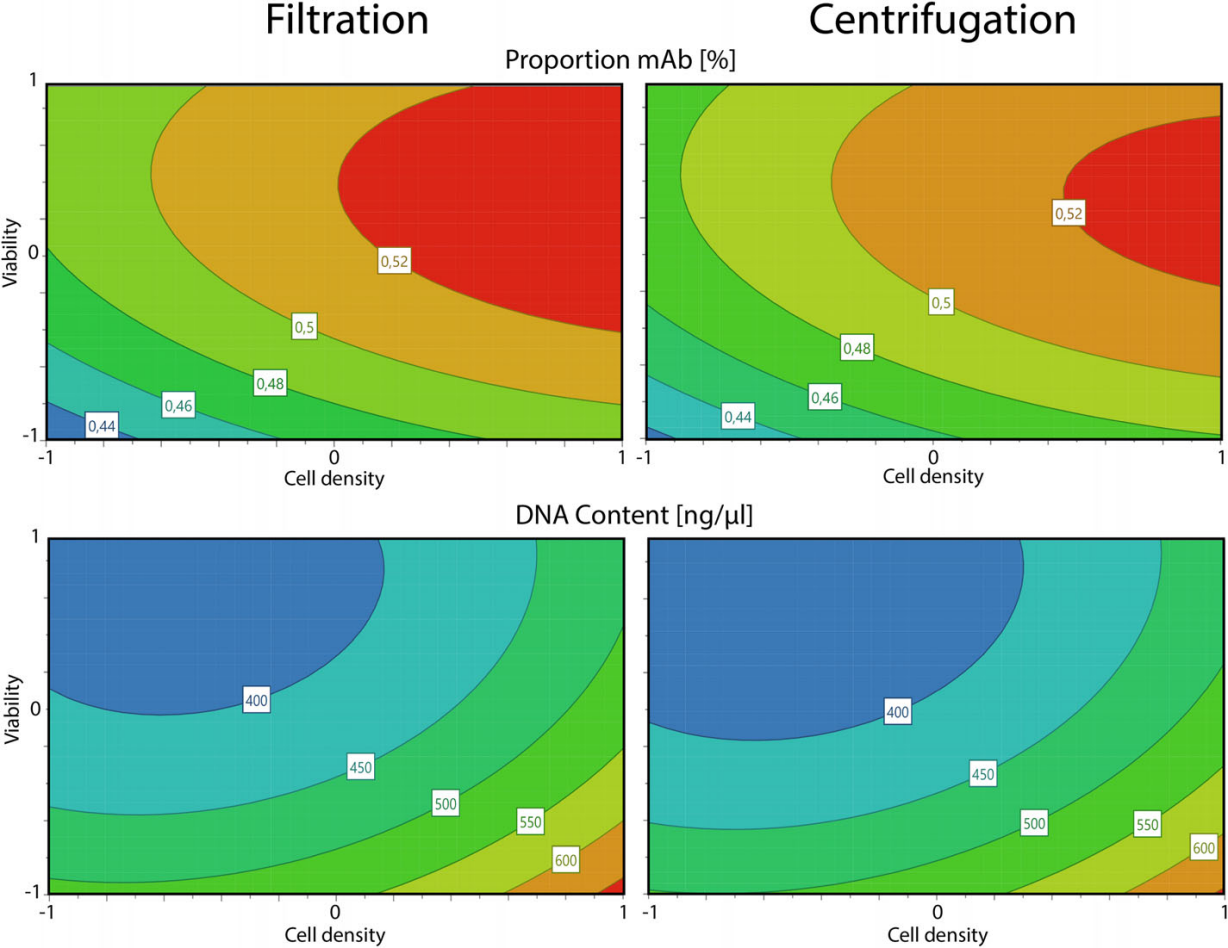
Response contour plots representing the interaction effects of factors viability and cell density on the studied process responses for the filtration (left) and the centrifugation (right).

The approved regression models were further used for the construction and analysis of response culture plots, which provide a lucid two‐dimensional evaluation of the factors and corresponding response values. Response plots for each response divided by the separation method are illustrated in Figure 5.

By analysis of the response contour plots the described factor effects and interactions were outlined more detailed. Comparison of the left and right plots confirms the marginal effect differences between the cell separation methods. For the mAb proportion the optimal area was only slightly enlarged using the filtration method. Overall, these differences can be rated as insignificant for the investigated responses, meaning the investigated cell separation methods have no critical effect on the primary recovery of the studied process.

**FIGURE 6:**
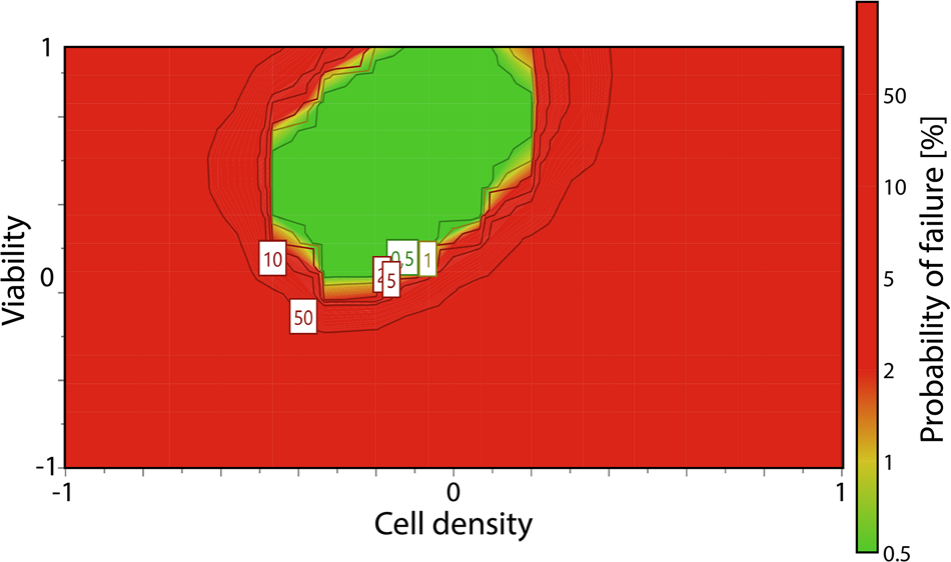
Determined design spaces for the mAb production process with color coded probability of failure for the assessed response specifications.

The factor effects of the viability and the cell density were also confirmed with the response contour plots. As expected, lower viabilities resulted in higher residual DNA concentrations as well as higher amounts of host cell proteins in the supernatant, thus a lower proportional mAb content. Apoptotic cells release large amounts of cell specific impurities to the cell culture medium, which are difficult to remove during the first steps of primary recovery. Removal of said impurities during the downstream process can be time consuming and costly, underlining the critical effect of the cell viability for the entire process performance. Higher cell densities during the cell separation improved the mAb proportion, while increasing the undesirable DNA content. Thereby the cell density shows a discrepancy between optimal mAb proportion and residual DNA. Both factors also showed interaction and non‐linearity effects. In order to further analyze the optimal factor set points and visualize the experimental design region in which all response specifications are fulfilled, a designated design space was calculated. Response specifications were accounted during the process development and adjusted during the risk assessment and data analysis. Figure 6 depicts the resulting design space, with green areas marking a robust design space with low probabilities for possible process failures.

The designated design space was established around 0 level for the cell density and between 0 and 1 level for the viability. Insignificant differences resulting from the separation methods were excluded during the design space determination. Summarized, the viability should be maintained above 85 % to avoid undesired amounts of HCPs and DNA impurities. In order to strike the balance between optimal proportions of the produced antibody to residual DNA the cell density during the cell removal should be controlled to be around 15 × 10^6^ cells/ml for the primary recovery of the studied fed batch process.

## 4 CONCLUDING REMARKS

Established Quality by Design principles were implemented in the first step of the primary recovery of a mAb production process. This study builds upon previous work on the inoculum's expansion as well as the production step and marks the final step toward a complete upstream process characterization following the FDA guidelines. Critical process parameters were determined during the risk assessment and combined in a DoE approach. In order to adjust the cell viability during the process without elongation of the culture duration, a directed cell death protocol using cytotoxic camptothecin was established. Thereby, it was possible to controllably vary the viability between 60%–99 %. The process was conducted using the ambr250 bioreactor platform, ensuring optimal process control for the desired scale. A mathematical model was calculated to fit the compiled response data using multivariate data analysis. By that, parameter significance was assessed and specific parameter effects and interactions were identified. Bioactivity of the produced antibody was confirmed to stay intact over the varied experimental runs, ruling out parameter effects on the mAb quality. The purity of the supernatant was assessed by measurement of HCPs and residual DNA amounts and used as critical quality attribute because of its crucial effect on the following downstream process. The comparison between centrifugation and filtration as separation methods did not result in significant changes in the amount of impurities in the supernatant. Cell viability and cell density during the separation were determined as non‐linear key process parameters with interaction effects.

These responses were used to establish a design space for optimal mAb proportion and low amounts of residual DNA after the primary recovery. As expected, maintenance of high cell viabilities was determined to be crucial to reduce undesired impurities. The cell density showed a contrary effect on the amounts of HCPs and DNA, with higher cell densities increasing the DNA content while lowering the concentration of HCPs. Therefore, the design space was calculated to combine optimal mAb proportion while maintaining DNA amounts under the defined limit. In conclusion, the viability should be maintained above 85% and the cell density should be controlled around 15 × 10^6^ cells/ml during the cell removal.

The described case study highlights the importance of cell maintenance during the entire upstream process. It confirms the previous findings that changes during the production part of the established mAb production process have no significant influence on the bioactivity of the antibody [19]. Thereby, process control and primary recovery should be tuned toward high purity of the supernatant, enabling time and cost efficient downstream processes.

## CONFLICT OF INTEREST

We confirm that all corresponding authors agree with the submission and publication of this paper and that there is no conflict of interest concerning financial and personal relationships. The manuscript does not contain neither experiments using animals nor human studies. Furthermore, we confirm that the article has not been published previously by any of the authors and is not under consideration for publication elsewhere at the time of submission.

## Supporting information

Supporting Information

## References

[1] El Abd Y , Tabll A , Smolic R , Smolic M . Mini‐review: the market growth of diagnostic and therapeutic monoclonal antibodies – SARS CoV‐2 as an example. Hum Antibodies. 2022;30:15‐24. 34958012 10.3233/HAB-211513

[2] Grilo AL , Mantalaris A . The increasingly human and profitable monoclonal antibody market. Trends Biotechnol. 2019;37:9‐16. 29945725 10.1016/j.tibtech.2018.05.014

[3] Jin S , Sun Y , Liang X , et al. Emerging new therapeutic antibody derivatives for cancer treatment. Signal Transduct Target Ther. 2022;7:39. 35132063 10.1038/s41392-021-00868-x PMC8821599

[4] Wang C , Li W , Drabek D , et al. A human monoclonal antibody blocking SARS‐CoV‐2 infection. Nat Commun. 2020;11:2251. 32366817 10.1038/s41467-020-16256-y PMC7198537

[5] Jahanshahlu L , Rezaei N . Monoclonal antibody as a potential anti‐COVID‐19. Biomed Pharmacother. 2020;129:110337. 32534226 10.1016/j.biopha.2020.110337 PMC7269943

[6] Santos‐Neto JF , Oliveira FO , Hodel KVS , et al. Technological advancements in monoclonal antibodies. Sci World J. 2021;2021:1‐19. 10.1155/2021/6663708 PMC7892242 33628140

[7] Snee RD . Quality by design: building quality into products and processes. In: Zhang L , ed. Nonclinical Statistics for Pharmaceutical and Biotechnology Industries. Springer International Publishing; 2016: 461‐499. doi: 10.1007/978-3-319-23558-5_18

[8] US Food and Drug Administration . Guidance for Industry PAT ‐ A Framework for Innovative Pharmaceutical Development, manufacturing, and Quality Assurance. 19 (2004).

[9] The International Conference of Harmonisation of Technical Requirements for Regristration of Pharmaceuticals for Human Use. ICH harmonised tripartie guideline: Pharmaceutical development Q8 (R2). 2009.

[10] The International Conference of Harmonisation of Technical Requirements for Regristration of Pharmaceuticals for Human Use. ICH harmonised tripartie guideline: Quality risk management Q9. 2005.

[11] The International Conference of Harmonisation of Technical Requirements for Regristration of Pharmaceuticals for Human Use. ICH harmonised tripartie guideline: Pharmaceutical quality systems Q10. 2008.

[12] US Food and Drug Administration . Pharmaceutical cGMPs for the 21st Century; a risk‐based approach. 2004.

[13] Rathore AS , Winkle H . Quality by design for biopharmaceuticals. Nat Biotechnol. 2009;27:9. 19131992 10.1038/nbt0109-26

[14] Rathore AS . QbD/PAT for bioprocessing: moving from theory to implementation. Curr Opin Chem Eng. 2014;6:1‐8.

[15] Rathore AS . Roadmap for implementation of quality by design (QbD) for biotechnology products. Trends Biotechnol. 2009;27:546‐553. 19647883 10.1016/j.tibtech.2009.06.006

[16] Aksu B , Sezer AD , Yegen G , Kusçu L . QbD implementation in biotechnological product development studies. In: Chen T & Chai SC , eds. Special Topics in Drug Discovery. InTech; 2016. doi: 10.5772/66296

[17] Harms J , Wang X , Kim T , Yang X , Rathore AS . Defining process design space for biotech products: case study of Pichia pastoris fermentation. Biotechnol Prog. 2008;24:655‐662. 18412404 10.1021/bp070338y

[18] Böhl OJ , Schellenberg J , Bahnemann J , et al. Implementation of QbD strategies in the inoculum expansion of a mAb production process. Eng Life Sci. 2021;21:196‐207. 33716618 10.1002/elsc.202000056 PMC7923587

[19] Wohlenberg OJ , Kortmann C , Meyer KV , et al. Optimization of a mAb production process with regard to robustness and product quality using quality by design principles. Eng Life Sci. 2022;22:484‐494. 35865649 10.1002/elsc.202100172 PMC9288990

[20] Nagashima H , Watari A , Shinoda Y , Okamoto H , Takuma S . Application of a quality by design approach to the cell culture process of monoclonal antibody production, resulting in the establishment of a design space. J Pharm Sci. 2013;102:4274‐4283. 24122699 10.1002/jps.23744

[21] Abu‐Absi SF , Yang L , Thompson P , et al. Defining process design space for monoclonal antibody cell culture. Biotechnol Bioeng. 2010;106:894‐905. 20589669 10.1002/bit.22764

[22] Bodley AL , Shapiro TA . Molecular and cytotoxic effects of camptothecin, a topoisomerase I inhibitor, on trypanosomes and Leishmania. Proc Natl Acad Sci. 1995;92:3726‐3730. 7731973 10.1073/pnas.92.9.3726 PMC42034

[23] Janoschek S , Schulze M , Zijlstra G , Greller G , Matuszczyk J . A protocol to transfer a fed‐batch platform process into semi‐perfusion mode: the benefit of automated small‐scale bioreactors compared to shake flasks as scale‐down model. Biotechnol Prog. 2019;35:e2757. 30479066 10.1002/btpr.2757 PMC6667907

[24] Popova D , Stonier A , Pain D , Titchener‐Hooker NJ , Farid SS . Integrated economic and experimental framework for screening of primary recovery technologies for high cell density CHO cultures. Biotechnol J. 2016;11:899‐909. 27067803 10.1002/biot.201500336 PMC4999028

