# Supplementary material for: Employing QbD strategies to assess the impact of cell viability and density on the primary recovery of monoclonal antibodies
Source: Eng Life Sci. 2022 Dec 23;23(2):e202200056. doi: 10.1002/elsc.202200056 (PMC9893750; doi:10.1002/elsc.202200056)
Supplement: Supplementary file 1 — Supporting Information [file ELSC-23-e202200056-s001.docx]

**Supplements – Employing a QbD approach to assess the impact of cell viability and density on the primary recovery of monoclonal antibodies**

**Table S1.** Numerical coding of varied process parameters.

| Process Parameter | -1 | 0 | 1 |
| --- | --- | --- | --- |
| Viability [%] | 64 | 82.5 | 99 |
| VCD [x10^6^ cells/ml] | 10 | 15 | 20 |
| Separation method | Filtration | - | Centrifugation |

**Table S2.** Experimental set up and responses for the DoE.

| Experiment number | Viability [%] | VCD  [x10^6^ cells/ml] | Separation method | mAb titer [g/L] | Total protein [g/L] | Proportion  mAb [%] | Residual  DNA [ng/µl] | Bioactivity  low mAb conc. [%] | Bioactivity  high mAb conc. [%] |
| --- | --- | --- | --- | --- | --- | --- | --- | --- | --- |
| 1 | 99 | 10 | Filtration | 2.45 | 5.09 | 0.482 | 392.5 | 70.8 | 109.5 |
| 2 | 99 | 20 | Filtration | 2.42 | 4.65 | 0.521 | 497.5 | 75.1 | 89.6 |
| 3 | 82.5 | 10 | Filtration | 2.45 | 5.19 | 0.472 | 421.4 | 61.1 | 97.1 |
| 4 | 82.5 | 15 | Filtration | 2.73 | 5.36 | 0.510 | 429.6 | 60,.5 | 97.5 |
| 5 | 82.5 | 15 | Filtration | 2.72 | 5.33 | 0.510 | 436.1 | 48.0 | 89.2 |
| 6 | 82.5 | 15 | Filtration | 2.74 | 5.33 | 0.514 | 433.7 | 69.2 | 97.8 |
| 7 | 82.5 | 20 | Filtration | 2.42 | 4.61 | 0.526 | 537.9 | 79.4 | 101.4 |
| 8 | 64 | 10 | Filtration | 1.97 | 5.15 | 0.384 | 587.8 | 61.4 | 96.6 |
| 9 | 64 | 20 | Filtration | 1.96 | 4.53 | 0.433 | 751.4 | 77.5 | 103.1 |
| 10 | 99 | 10 | Centrifugation | 2.43 | 5.19 | 0.467 | 380.4 | 65.7 | 104.8 |
| 11 | 99 | 20 | Centrifugation | 2.41 | 4.66 | 0.518 | 480.7 | 69.5 | 91.1 |
| 12 | 82.5 | 10 | Centrifugation | 2.45 | 5.27 | 0.465 | 420.5 | 72.1 | 90.4 |
| 13 | 82.5 | 15 | Centrifugation | 2.70 | 5.38 | 0.501 | 412.0 | 62.3 | 83.6 |
| 14 | 82.5 | 15 | Centrifugation | 2.70 | 5.39 | 0.500 | 429.5 | 48.7 | 91.1 |
| 15 | 82.5 | 15 | Centrifugation | 2.67 | 5.38 | 0.497 | 428.6 | 71.9 | 98.3 |
| 16 | 82.5 | 20 | Centrifugation | 2.40 | 4.64 | 0.518 | 536.5 | 82.1 | 85.3 |
| 17 | 64 | 10 | Centrifugation | 1.97 | 5.22 | 0.378 | 565.0 | 63.6 | 88.1 |
| 18 | 64 | 20 | Centrifugation | 1.98 | 4.44 | 0.445 | 745.1 | 63.9 | 98.0 |

**Table S3.** Parameter limits for the design space calculation

|  | Proportion mAb [%] | Residual DNA [ng/µl] |
| --- | --- | --- |
| Parameter limit | 0.5 | 420 |
| Target | 1 | 0 |

**Table S4.** Used Cedex Bio kits for the metabolite analysis

| Metabolite | Cedex kit |
| --- | --- |
| Glucose | Glucose Cedex Bio |
| Lactate | Lactate Cedex Bio |
| Glutamine | Glutamine V2 Bio |
| Glutamate | Glutamate V2 Bio |
| Total protein | Total Protein Bio |
| Monoclonal antibody | IgG Bio |

**Table S5.** Camptothecin standards

| CPT concentration [µM]  / incubation time [h] | Proportion of apoptotic cells [%] | Standard deviation [%] |
| --- | --- | --- |
| 5 µM / 24 h | 3.4 | 0.68 |
| 5 µM / 48 h | 13.3 | 2.31 |
| 10 µM / 24 h | 7.5 | 1.58 |
| 10 µM / 48 h | 20.9 | 2.38 |
| 20 µM / 24 h | 11.8 | 2.13 |
| 20 µM / 48 h | 27.6 | 3.04 |
| 30 µM / 24 h | 15.7 | 1.97 |
| 30 µM / 48 h | 38.2 | 2.93 |

**Equation S1.** MLR equation for MVDA

$$R_{i}=p_{0}+p_{1}F_{1}+p_{2}F_{2}+p_{3}F_{3}+p_{4}F_{1}^{2}+p_{5}F_{2}^{2}+p_{6}F_{3}^{2}+p_{7}F_{1}F_{2}+p_{8}F_{1}F_{3}+p_{9}F_{2}F_{3}$$

Here, $R_{i}$ is one of the four responses, p_0_ is the intercept, p_j_ are the coefficients of the factors F_1_, F_2_ and F_3_. Factors whose coefficient has the value zero in its confidential interval are regarded to have no significant influence on the response and are therefore removed from the model.
